# Applying Satyrization to Insect Pest Control: The Case of the Spotted Wing Drosophila, *Drosophila suzukii* Matsumura

**DOI:** 10.3390/insects14060569

**Published:** 2023-06-19

**Authors:** Flavia Cerasti, Valentina Mastrantonio, Romano Dallai, Massimo Cristofaro, Daniele Porretta

**Affiliations:** 1 Department of Environmental Biology, Sapienza University of Rome, 00185 Rome, Italy; flavia.cerasti@uniroma1.it (F.C.); daniele.porretta@uniroma1.it (D.P.); 2 Department of Life Sciences, University of Siena, Via A. Moro 2, 53100 Siena, Italy; romano.dallai@unisi.it; 3 Biotechnology and Biological Control Agency (BBCA), 00123 Rome, Italy; m.cristofaro55@gmail.com

**Keywords:** interspecific interactions, insect pests, biological control, reproductive interference, asymmetric fitness cost, fruit fly

## Abstract

**Simple Summary:**

Satyrization, a form of sexual interaction between males of one species with females of another species, has attracted renewed interest in pest management strategies. By inducing fitness costs in one or both interacting species, satyrization may indeed dramatically affect population dynamics, being a valuable tool to be used alone, or in conjunction with other area-wide control approaches. Here, we aimed to investigate the potential use of satyrization to control the invasive pest *Drosophila suzukii* by using *D. melanogaster* males. By realizing courtship tests, spermathecae analysis, and multiple-choice experiments, we showed that *D. melanogaster* males were able to successfully court, mate and reduce the offspring of *D. suzukii* females. These results, overall, showed that the use of *D. melanogaster* males can be an effective tool to control *D. suzukii* and lay promising foundations for testing the application of this approach in field conditions.

**Abstract:**

*Drosophila suzukii* represents one of the major agricultural pests worldwide. The identification of safety and long-lasting tools to suppress its populations is therefore crucial to mitigate the environmental and economic damages due to its occurrence. Here, we explore the possibility of using satyrization as a tool to control the abundance of *D. suzukii*. By using males of *D. melanogaster*, we realized courtship tests, spermathecae analysis, and multiple-choice experiments to assess the occurrence and extent of pre- and post-zygotic isolation between the two species, as well as the occurrence of fitness costs in *D. suzukii* females due to satyrization. Our results showed that: (i) *D. melanogaster* males successfully courted *D. suzukii* females; (ii) *D. melanogaster* males significantly affected the total courtship time of *D. suzukii* males, which reduced from 22.6% to 6.4%; (iii) *D. melanogaster* males were able to inseminate *D. suzukii* and reduce their offspring, inducing a high fitness cost. Reproductive interference occurs at different steps between *D. melanogaster* and *D. suzukii*, both alone and in combination with other area-wide control approaches.

## 1. Introduction

Satyrization consists of reproductive interactions between individuals of different animal co-generic species and/or subspecies, which results in fitness costs for one or both the interacting individuals [1,2,3,4]. It results from incomplete mating barriers between species and can occur at any stage of mate acquisition throughout different mechanisms, from courtship to mating [2,3,5].

Satyrization has been documented in a wide variety of insect taxa under laboratory and field conditions [3]. Compelling examples suggest that satyrization can significantly affect the population dynamics of the interacting species with effects on species persistence or exclusion. For instance, in bean weevils *Callosobruchus maculatus* F. and *C. chinensis* L., reproductive interference is the critical factor determining species exclusion. In this system, behavioral experiments under laboratory conditions showed that males of neither species discriminated between conspecific and heterospecific females. However, *C. chinensis* showed more frequent behavioral interference than *C. maculatus* males, reducing the fecundity and longevity of heterospecific females and leading to *C. maculatus* exclusion [6]. Similarly, asymmetric satyrization has been documented in *Tribolium castaneum* Herbst and *T. confusum* Jacquelin du Val, due to the asymmetric promiscuity of males of the two species. Contrary to *T. castaneum* males, *T. confusum* males indiscriminately attempt to copulate with females of both species in laboratory assays [7]. Furthermore, *T. confusum* males damage the genitalia of *T. castaneum* females. This asymmetric satyrization reduces the fecundity and longevity of *T. castaneum* females, which depends on the frequency of *T. confusum* males [8]. Asymmetric mating interactions have been observed in nature between different species pairs, including native and invasive taxa [2,3]. For example, in the whitefly *Bemisia tabaci* Gerradius, long-term field surveys, caged population experiments, and behavioral tests supported a significant role of satyrization in driving the invasion of the B-biotype in China and Australia and the displacement of the native biotype [9].

Because of its dramatic effects on population dynamics, satyrization can be a valuable tool for pest control. This approach was proposed some decades ago [1,10], but only recently has it sparked a renewed interest from the scientific community [5]. Honma et al. [11] have proposed a framework for the incorporation of satyrization into a sterile insect program. Likewise, Mitchell et al. [5] reviewed the literature on interspecific mating interactions, addressed mechanisms and outcomes, and outlined a framework for using satyrization in pest control. In particular, two forms of satyrization have been proposed as interesting in pest control: one form that uses the release of both sexes of the interfering species to replace the pest population, and the other one that uses the release of just one sex of the interfering species to reduce or eliminate the pest population [5,12,13].

Here, we aimed to investigate the possible use of one-sex satyrization to control *Drosophila suzukii* Matsumura (Diptera: Drosophilidae) using males of *D. melanogaster* Meigen. *Drosophila suzukii* is an invasive species that has spread in the last few decades from its native range in East Asia throughout North America, Europe, and South America [14]. Unlike most Drosophilidae, *D. suzukii* can lay eggs in unripe and healthy fruits, causing severe economic losses for fruit industries worldwide [15,16]. Laboratory observations have suggested courtship and mating interference between males and females of *D. suzukii* and *D. melanogaster* [17]. However, the occurrence and extent of satyrization as well as its potential use to control *D. suzukii* remain unexplored.

In this paper, we specifically aimed to assess: (i) the occurrence and extent of pre-zygotic isolation between *D. suzukii* and *D. melanogaster*. To this end, we carried out courtship and mating tests (experiments 1 and 2); (ii) the extent of post-zygotic isolation between the two species. To this end, we analyzed *D. suzukii* spermathecae of females mated with *D. melanogaster* males (experiment 3); (iii) whether the satyrization by *D. melanogaster* males leads to a fitness cost for *D. suzukii* females. To this end, we analyzed the effect of *D. melanogaster* males on the fertility of *D. suzukii* using different species ratios (experiment 4).

## 2. Materials and Methods

*Drosophila suzukii* and *D. melanogaster* laboratory colonies were established at Sapienza University of Rome. *Drosophila suzukii* adults were collected from infested cherry orchards at San Michele all’Adige (Trento, Italy); adults of colony of *D. melanogaster* were provided by the Laboratory Agrifood Sustainability, Quality and Safety of ENEA Casaccia Research Centre (Rome). The collected individuals were recognized using diagnostic morphological traits that univocally discriminate the two species [18], and DNA barcoding using the Cytochrome Oxidase I (COI) gene [19]. The colonies were maintained separated in entomological cages (30 × 30 × 30 cm) in a walk-in climate chamber at 25 ± 1 °C, 14:10 h light:dark cycle, and fed with a cornmeal diet (89% dH_2_O, 0.6% Fisher agar, 1.4% table sugar, 6.3% precooked ground maize, 1.5% mother yeast, 1% soy flour and 0.2% methylparaben, dissolved in 25 mL of 70% ethanol). Adults had unrestricted access to water. 

### 2.1. Experiment 1: Analysis of Courtship Behavior between D. suzukii and D. melanogaster

To assess the occurrence and extent of pre-zygotic isolation between the two species, we evaluated if *D. melanogaster* males were able to court *D. suzukii* females and affect the courtship rate of *D. suzukii* males. We obtained virgin males and females of the two species by checking pupae every thirty minutes, collecting newly emerged individuals as soon as they emerged, and placing males and females in separate cages. We used seventy-two hours old virgin individuals to ensure they were sexually mature [20,21]. Three different courtship tests were carried out in plastic falcons (15 mL) as follows: (1) one *D. melanogaster* male was confined with one *D. suzukii* female; (2) one *D. melanogaster* male and one *D. suzukii* male were confined with one *D. suzukii* female; (3) one *D. suzukii* male was confined with one *D. suzukii* female. We recorded a 10 min video for each condition with an Olympus Tough TG-6 camera. In each test, we recorded the typical courtship behaviors of the *D. melanogaster* and *D. suzukii* male [20,21]. In test 2, we analyzed the courting behavior of both males. In all tests, we compared the time spent by males in courting the *D. suzukii* females. Twenty-two replicates for each test were carried out.

### 2.2. Experiment 2: Analysis of Insemination between D. suzukii and D. melanogaster

The occurrence and extent of pre-zygotic isolation between the two species was also investigated carrying out no-choice experiments to assess if *D. melanogaster* males can inseminate *D. suzukii* females. First, virgin *D. suzukii* females and *D. melanogaster* males were selected, using the method described above [20,21]. Then, 5 *D. suzukii* females and 10 *D. melanogaster* males (72 h old) were placed in a plastic falcon (50 mL) containing 15 mL of food substrate. After 48 h, the females were removed, and their mating status was determined by detecting the presence of sperm within spermathecae, which preserve sperm for a longer time than seminal receptacle [22]. Female genital apparatuses were dissected under a light microscope in a phosphate-buffered solution 0.1 M pH 7.2, to which 3% of sucrose was previously added. After the extraction of spermathecae, these were placed in a drop of buffer solution, covered with a coverslip, and squashed with a light pressure. The preparations were observed with a Leica DMRB light phase-contrast microscope. Five replicates were carried out, and a total of 25 females were dissected.

### 2.3. Experiment 3: Analysis of Larval Development Resulting from Insemination by D. melanogaster

We investigated the occurrence of post-zygotic isolation between the two species, provided that *D. melanogaster* males can inseminate *D. suzukii* females (see Section 3). One virgin *D. suzukii* female and one virgin *D. melanogaster* male were placed in a plastic falcon (50 mL) containing 15 mL of food substrate for female oviposition and larval development. The couples were maintained in the falcon for six days to allow mating and eggs deposition [20,21]. Then, the adults were removed, and the food substrate in each falcon was checked to find eggs, using a stereomicroscope Leica EZ4W at magnification 5×. If present, eggs were photographed with stereomicroscope digital camera and monitored for eclosion and larval development. Thirty-four replicates were carried out.

### 2.4. Experiment 4: Analysis of Satyrization of D. melanogaster Males on the Fertility of D. suzukii

To assess the impact on *D. suzukii* fitness of satyrization by *D. melanogaster* males, we compared the offspring of *D. suzukii* with and without *D. melanogaster* males. Five pairs of *D. suzukii* adults (virgin males and females) were placed in entomological cages (15 × 15 × 15 cm) with 0, 20, 40, 60 *D. melanogaster* males. A plastic falcon (50 mL) containing 15 mL of food substrate was placed in each cage for female oviposition and larval development. After six days, the falcons were removed from the cages, and the number of offspring that emerged from each cage was counted and then compared among conditions. The cages were maintained under the same conditions as the colonies, and five replicates for each treatment were carried out. 

### 2.5. Data Analysis

For experiment 1, the time spent by each male in each courtship element and the total time spent by males in courting were recorded using the “BORIS” Behavior Analysis Program [23]. The Wilcoxon Mann–Whitney U test was used to compare the time spent courting by *D. melanogaster* and *D. suzukii* males using the R software vers.4.1.2 (http://www.R-project.org/, accessed on 15 June 2023). For experiment 2, we checked the *D. suzukii* spermathecae as described in Section 2.2 and calculated the percentage of spermathecae with sperms. For experiment 3, we checked the experimental food substrates to find eggs and calculated the percentage of the substrates with eggs. For experiment 4, we performed a generalized linear model (GLM), then used a post hoc Tukey’s multiple comparison test to assess the effect of the *D. melanogaster* males on *D. suzukii* offspring. The analyses were performed using the *ghlt* function implemented in *multcomp* R-package [24].

## 3. Results

### 3.1. Experiment 1: Courtship Behavior between D. suzukii and D. melanogaster

**Figure 1:**
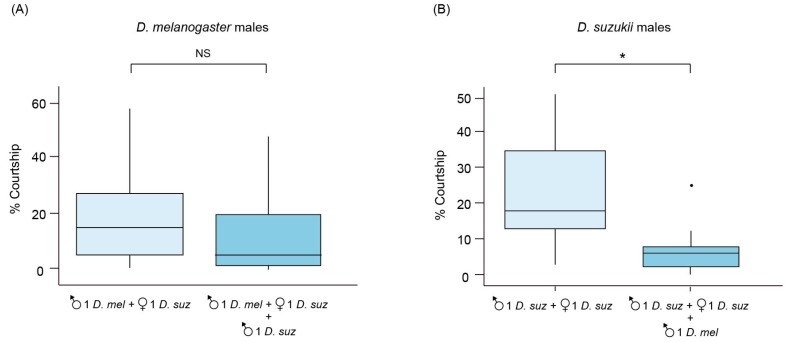
Time budget of courtship behavior of *D. suzukii* and *D. melanogaster* males. (**A**) Percentage of the total time spent courting *D. suzukii* females by *D. melanogaster* males without *D. suzukii* males (light pale blue) and with *D. suzukii* males (dark pale blue). (**B**) Percentage of the total time spent courting *D. suzukii* females by *D. suzukii* males without *D. melanogaster* males (light pale blue) and with *D. melanogaster* males (dark pale blue). Black dot is box-plot outlier. Asterisk means Wilcoxon Mann–Whitney test *p*-value < 0.05.

In courtship experiments, we investigated if *D. melanogaster* males were able to court *D. suzukii* females and affect the courtship of *D. suzukii* males. We found that both *D. suzukii* and *D. melanogaster* males showed typical behavior elements during courtship under all the experimental conditions, including “orientation” (i.e., the male approaches the female, quivering the abdominal and scissoring its wings), “tapping,” (i.e., the male hits the female abdomen, or middle and hind legs by stretching his foreleg); “wing spreading”, “wing scissoring” (i.e., the male is oriented toward the female front, quivers with the abdomen and scissors his wings keeping them at 180° for seconds to expose the upper side and wing spot toward to female) (Table 1). The total time spent by *D. melanogaster* males in courting *D. suzukii* females was 18.17% (±2.97) (mean ± standard error) when they were alone, and 10.96% (±2.80) when they were with *D. suzukii* males. No significant differences were observed between the two conditions (Wilcoxon Mann–Whitney test W = 422.5, *p*-value = 0.050) (Figure 1A). The total time spent by *D. suzukii* males courting *D. suzukii* females was 22.64% (±3.13) when they were alone, and it was significantly reduced (6.42% ± 1.37) when they were placed with *D. melanogaster* males (Wilcoxon Mann–Whitney test W = 127, *p*-value = 0.029) (Figure 1B). 

### 3.2. Experiment 2: Insemination between D. suzukii and D. melanogaster

To assess if *D. melanogaster* males were able to inseminate *D. suzukii* females, we analyzed the content of spermathecae dissected from virgin *D. suzukii* females that had been placed with *D. melanogaster* males (for 48 h). We found that 20 out of 25 females (80%) showed spermathecae with sperms.

### 3.3. Experiment 3: Larval Development after Insemination by D. melanogaster

Post-zygotic isolation between *D. suzukii* females and *D. melanogaster* males was assessed by analyzing if eggs were oviposited by *D. suzukii* females confined with *D. melanogaster* males to mate, and if larvae developed after egg-hatching. We found eggs in 7 out of 34 (21%) oviposition/food substrates. No larvae were observed in any oviposition/food substrates.

### 3.4. Experiment 4: Effect of Satyrization of D. melanogaster Males on the Fertility of D. suzukii

**Figure 2:**
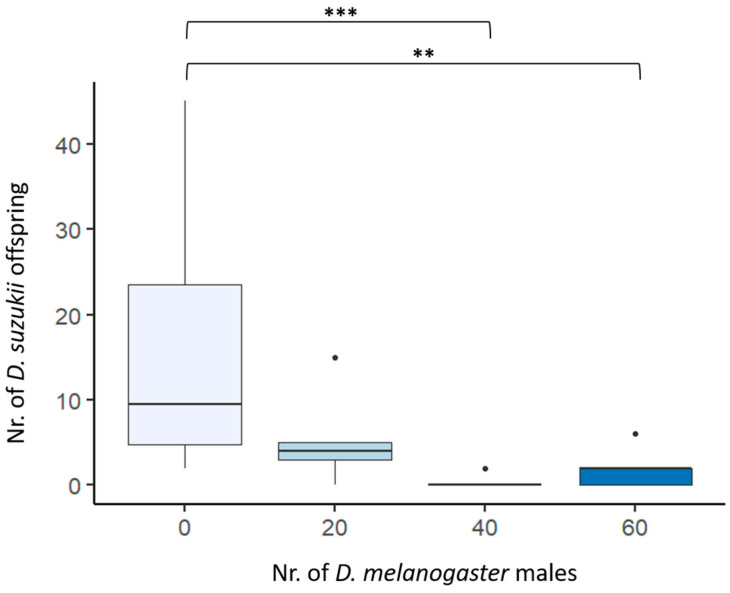
Offspring originated from five pairs of *Drosophila suzukii* males and females with 0, 20, 40 or 60 *D. melanogaster* males. *** Wilcoxon Mann–Whitney test *p* < 0.001; ** Wilcoxon Mann–Whitney test *p*-value < 0.01. Black dots are box-plot outliers.

The presence of *D. melanogaster* males significantly reduced the number of *D. suzukii* offspring. The mean number (±standard error) of the offspring originated from five pairs of *Drosophila suzukii* males and females with 0, 20, 40, or 60 *D. melanogaster* males were 15.5 (±4.11), 5.4 (±2.54), 0.4 (±0.4), and 2.0 (±1.09), respectively. The GLM showed a significant effect of the number of *D. melanogaster* males on the number of offspring produced by *D. suzukii* females (F_3,23_ = 3.778 *p*-value = 0.024). The post hoc Tukey’s tests showed that the highest reduction occurred when 40 (z = −4.300, *p*-value < 0.001) and 60 (z = −3.602, *p*-value = 0.01) *D. melanogaster* males were present (Figure 2). 

## 4. Discussion

This paper aimed to investigate the potential application of satyrization to control *D. suzukii* by using *D. melanogaster* males. *D. melanogaster* satisfies critical factors for the application of the satyrization approach [5]. The first concern in applying satyrization is the risk of introducing non-native or pest species. This is not the case with *D. melanogaster.* It is a cosmopolitan species occurring in sympatry with *D. suzukii* in many invaded areas [25,26]. Furthermore, it is not an agricultural pest as the female oviposits on rotten fruits [27]. 

Second, the potential use of satyrization as a control method strictly depends on the occurrence/extent of pre-mating and post-mating barriers between the target and the control species. Our results supported incomplete pre-zygotic isolation between *D. suzukii* and *D. melanogaster*. Behavioral tests showed that *D. suzukii* females were indeed courted by *D. melanogaster* as much as *D. suzukii* males. Most importantly, the total courtship time by *D. suzukii* males decreased when *D. melanogaster* males were co-occurring. We did not observe interspecific copulation during the ten-minute courtship experiments. However, the analysis of spermathecae in virgin *D. suzukii* females showed that *D. melanogaster* males were able to inseminate *D. suzukii* females. Contrary to pre-zygotic isolation, we found that post-zygotic isolation between *D. suzukii* and *D. melanogaster* is complete. Indeed, only a few *D. suzukii* females oviposited eggs, and no larval development was observed in any tests. Therefore, reproductive interference between the two species occurs at different steps, not only through courtship but also through copulation and hybridization.

Third, for successful control by satyrization, the interfering species must lead fitness costs to the target species. Our results satisfied this condition, showing that *D. suzukii* couples had significantly reduced offspring in the presence of *D. melanogaster* males (Figure 2). Fertility reduction of *D. suzukii* females has been suggested from some authors to be due to chemical interference by *D. melanogaster* during mating. Indeed, it has been shown in most *Drosophila* species, including *D. melanogaster*, that the cis-vaccenyl acetate (cVA) pheromone, produced by males during courtship, has a disruptive effect on *D. suzukii*, resulting in reducing mating as a natural repellent to *D. suzukii* female for laying eggs [28,29]. However, our results of behavioral tests, pre- and post-zygotic barriers tests, and the density-dependent fitness cost observed support the idea that satyrization is a major driver of the fitness cost of *D. suzukii*. Because of the interference of *D. melanogaster* males, *D. suzukii* males would indeed court females for less time, be disturbed, or fail to fertilize females [4,30]. Interestingly, *D. melanogaster* males release substances through seminal fluid that reduce female remating in homospecific matings [31,32]. If such a phenomenon also occurs in heterospecific matings between *D. suzukii* and *D. melanogaster*, the fitness cost due to satyrization by *D. melanogaster* males would also be higher.

Following the framework of Mitchell et al. [5], other factors should be addressed in considering the application of satyrization in pest control. For example, the females of the target species could evolve resistance to heterospecific mating by reproductive character displacement. This process has been documented in nature in studies on speciation by reinforcement. Under reinforcement, because of the fitness costs due to heterospecific mating, natural selection affects the components of the mate recognition system, leading them to diverge and complete pre-mating isolation between the two interbreeding taxa [33,34]. However, it has been argued that resistance could not be a major problem for sterile interference programs. The occurrence of reinforcement in nature is indeed limited to specific conditions, and reproductive character displacement takes a longer time to occur than that for species exclusion during a sterile interference program [35,36,37]. Furthermore, both modelling and field studies of sterile insect technique (SIT) showed that by releasing enough sterile males, the effect of female resistance can be overcome [38,39]. Despite these arguments, reproductive character displacement has been documented in *Drosophila* species [40], and the possible resistance evolution in *D. suzukii* females should deserve attention in future studies aimed to apply satyrization programs.

Possible interference with other control approaches should also be considered [5]. For example, reproductive interference has been observed under laboratory conditions between *Eretmocerus mundus* AUTH males and *E. eremicus* AUTH females, two parasitoid species of the whitefly *B. tabaci*. Therefore, the effectiveness of the biological control could be negatively affected in areas where both species co-occur [41]. On the other hand, satyrization can synergize with other control methods, leading to more effective control of the target species. Honma et al. [11]. have recently proposed a combined application of SIT and satyrization (“sterile interference”), arguing that sterile insects could be used to suppress the wild population of the same species (under a classic SIT program), and that of a co-occurring closely related species by reproductive interference. 

Currently, numerous strategies are used or are being explored to control *D. suzukii* worldwide [25,42]. In addition to cultural control approaches and field sanitation, control strategies include chemical control using synthetic or natural insecticides; biological control using parasitoids, predators, pathogens, and entomopathogenic organisms; autocidal control using SIT and incompatible insect technique (IIT); biotechnology-based strategies, including gene silencing and genome editing approaches. SIT has provided encouraging results in recent years [38,39,43,44].

Satyrization under natural condition between wild *D. melanogaster* males and *D. suzukii* individuals has not been investigated. Both species coexist during the growing season in some geographic areas [45,46], although oviposition preferences could limit the encounters, contrary to laboratory conditions where they forcibly co-occur [47,48]. In conclusion, our results show the occurrence of reproductive interference between *D. suzukii* and *D. melanogaster* males, with high fitness costs for *D. suzukii*. They are promising for testing the effects of satyrization under field conditions. Massive release of *D. melanogaster* males could be an effective control approach, potentially also in conjunction with *D. suzukii* sterile males in SIT programs. In this context, it would also be interesting to test the effect of sterilized *D. melanogaster* males on *D. suzukii* (i.e., heterospecific SIT approach).

## Figures and Tables

**Table 1:** Percentage (±standard error) of courtship elements of *Drosophila melanogaster* and *D. suzukii* males without and with heterospecific males during 10 min of the testing period. *D. mel.* = *D. melanogaster*; *D. suz.* = *D. suzukii*.

Courtship Elements	Courtship Behavior of *D. melanogaster* Males		Courtship Behavior of *D. suzukii* Males	
	1 ♂*D. mel.* + 1 ♀ *D. suz.*	1 ♂*D. mel.* + 1 ♀ *D. suz.* + 1 ♂*D. suz*	1♂ *D. suz.* + 1♀ *D. suz.*	1♂ *D. suz.* + 1 ♀ *D. suz.* + 1♂ *D. mel.*
Orientation	1.28 (±0.44)	0.74 (±0.20)	0.03 (±0.02)	0.41 (±0.15)
Tapping	3.14 (±0.72)	1.60 + (±0.45)	4.20 (±2.32)	0.77 (±0.31)
Wing spreading	13.42 (±2.40)	8.46 (±2.54)	20.82 (±3.26)	5.70 (±1.56)
Wing scissoring	0.29 (±0.12)	0.15 (±0.06)	0.52 (±0.52)	0.09 (±0.05)

## Data Availability

All data are contained within the article.
